# HIV Infection among Illegal Migrants, Italy, 2004–2007

**DOI:** 10.3201/eid1511.090908

**Published:** 2009-11

**Authors:** Maria Chiara Pezzoli, Issa El Hamad, Carmelo Scarcella, Francesco Vassallo, Fabrizio Speziani, Graziella Cristini, Carla Scolari, Barbara Suligoi, Anna Maria Luzi, Daniela Bernasconi, Miriam Lichtner, Giuseppina Cassara’, Nino Manca, Giampiero Carosi, Francesco Castelli, the PRISHMA Study Group

**Affiliations:** Local Health Authority, Brescia, Italy (M.C. Pezzoli, C. Scarcella, F. Vassallo, F. Speziani, C. Scolari); Spedali Civili General Hospital, Brescia (I. El Hamad, G. Cristini); National Institute of Health, Rome, Italy (B. Suligoi, A.M. Luzi, D. Bernasconi); University La Sapienza, Rome (M. Lichtner); Casa del Sole Hospital, Palermo, Italy (G. Cassara’); University of Brescia, Brescia (N. Manca, G. Carosi, F. Castelli)

**Keywords:** HIV/AIDS and other retroviruses, viruses, migrants, prevalence, exposure, Italy, sub-Saharan Africa, dispatch

## Abstract

To determine HIV prevalence and place of exposure for illegal migrants in Italy, we tested 3,003 illegal adult migrants for HIV; 29 (0.97%) were HIV positive. Antibody avidity index results (indicators of time of infection) were available for 27 of those persons and showed that 6 (22.2%) presumably acquired their infection after migration.

During the past 2 decades, Italy has had an uncontrolled increase in number of migrants. As many as 4 million (720,000 undocumented) foreign-born persons (≈7% of the population in Italy) are living in Italy (62.5% in the northern region, 25% in the central region, and 12.5% in the southern region) (*1*).

Sexually transmitted infections (STIs) are present in migrants, especially early after migration. Migrants are usually men (single or married) who live alone. Migrant women are often forced into prostitution. These factors may expose them to risky sexual contacts and STIs. Preventive educational campaigns rarely reach migrant communities because of logistic, cultural, and language barriers.

As of December 2008, a total of 60,346 AIDS cases were reported in Italy, where the opt-in strategy (persons need to accept testing) of HIV testing is applied (*2*). The proportion of migrants among persons with cases of AIDS in Italy has progressively increased from 2.5% before 1993 to 20.7% in 2007–2008 (*2*). This finding reflects the increasing number of migrants and their delayed access to screening and medical care (*3*). The proportion of migrants among persons with new cases of HIV infection has also increased from 11% in 1992 to 32% in 2007 in Italy (*2*), a finding that confirms previous data (*4*). Estimated HIV incidence rates among migrants are 64.0 cases (for men) and 52.5 cases (for women)/100,000 persons in 2007. These rates are 11× higher than the HIV incidence rate for native-born Italians (*5*). No reliable information is available on the prevalence of HIV infection in the illegal migrant population in Italy. Data are available only for selected risk groups such as female commercial sex workers (*6*), transsexuals (*7*), and patients with STIs (*8*). Furthermore, no reliable data are available for likely place of infection for persons recently screened and found to be HIV infected. To address these issues, we determined the prevalence and likely place of infection with HIV for an illegal migrant population in Italy.

## The Study

The study protocol was reviewed and approved by the ethical boards of all participating centers. The study was conducted during January 2004–December 2007 in clinical centers that offered primary healthcare to illegal migrants in northern (Brescia), central (Rome), and southern (Palermo) Italy. All adult migrants from a non–European Union country who registered for a visit at each center were asked to participate in the study. Participants were divided into 4 groups according to their risk for HIV infection: 1) commercial sex workers, 2) persons reporting unsafe sex (occasional not-for-money homosexual or heterosexual contacts with persons other than their regular partner), 3) persons with other risk factors (intravenous drug use or blood transfusion in their country of origin, and 4) persons with no risk factors identified.

Participants were tested for antibodies against HIV types 1 and 2 and for p24 antigen by using a commercial microparticle enzyme immunoassay (AxSYM HIV Ag/Ab Combo; Abbott Laboratories, Abbott Park, IL, USA). HIV-positive serum samples were further tested for HIV antibody avidity by using the AxSYM HIV 1/2gO assay (Abbott Laboratories) as reported (*9*), to ascertain likely time of infection. An avidity index <0.80 indicated that infection was acquired recently (within the past 6 months); a higher index indicated that infection was acquired earlier (>6 months ago). A cutoff value of 0.80 was used and validated as having 93.0% sensitivity, 98.5% specificity (*10*), and >90.0% reproducibility. Avidity results were cross-checked with reported time of migration to assess likely place of exposure.

A total of 4,078 persons were invited to participate in the study. Of 3,976 (97.5%) who agreed to participate, 3,003 (73.6%) underwent HIV testing (2,815 in Brescia, 48 in Rome, and 140 in Palermo). In terms of HIV risk for 2,853 respondents, 191 were commercial sex workers, 1,246 practiced unsafe sex, 47 reported other risks, and 1,494 reported no risk factors (total = 2,978 because multiple risk factors were reported by some persons). Demographic characteristics for the participants are shown in Table 1.

**Table 1:** Sociodemographic characteristics of 3,003 migrants, Italy, 2004–2007

Characteristic	Value*
Sex	
F	1,587 (52.8)
M	1,416 (47.2)
Median age, y (range)	31 (18–74)
Marital status	
Married	1,236 (43.3)
Single	1,617 (56.7)
Place of origin	
Europe	1,341 (44.7)
Sub-Saharan Africa	674 (22.4)
Asia	470 (15.7)
North America	339 (11.3
Latin America	179 (6.0)
Median migration period, mo (range)	23.4 (0.2–324)
Religion†	
Christianity	1,854 (61.8)
Islam	901 (30.0)
Other	115 (3.8)
None	129 (4.3)
Education, y‡	
<8	775 (25.9)
>8	2,222 (74.1)
Job status	
Employed	1,545 (51.4)
Unemployed	1,458 (48.6)
Immigration status	
Illegal	2,748 (91.5)
Legal	255 (8.5)

*Except where indicated, values are no. (%). †n = 2,999. ‡n = 2,997

HIV-1 infection was detected for 29 (0.97%) of 3,003 participants (95% confidence interval [CI] 0.90%–1.2%); no participants were infected with HIV-2. Avidity index results were obtained for 27 of the 29 HIV-positive participants. Univariate analysis showed that sociodemographic and behavior factors associated with HIV infection were Christian religion (p = 0.029, odds ratio [OR] 3.07, 95% CI 1.06–8.83), migration from sub-Saharan Africa (p = 0.001, OR 11.94, 95% CI 1.61–88.81), commercial sex (p = 0.0001, OR 18.2, 95% CI 6.25–52.97), and unsafe sex (p = 0.016, OR 3.43, 95% CI 1.22–9.66). Multiple logistic regression showed that factors independently associated with increased risk for HIV infection were migration from sub-Saharan Africa (p = 0.0001, OR 3.7, 95% CI 2.2–9.4), commercial sex (p = 0.025, OR 18.4, 95% CI 4.9–48.5), and unsafe sex (p = 0.01, OR 2.3, 95% CI 1.7–8.6).

Place of infection could not be determined for 17 (63.0%) of 27 persons; 6 (22.2%) of 27 were presumably recently infected in Italy, and 4 (14.8%) of 27 presumably acquired their infection in their country of origin before emigration. Results of avidity index determinations are shown in the Figure and Table 2. Sociodemographic characteristics of our population did not differ from those reported nationwide (*1*).

**Figure:**
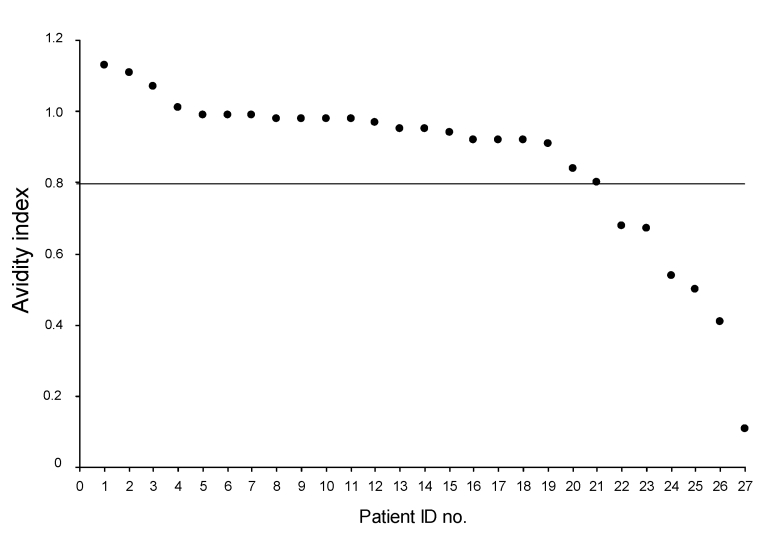
Antibody avidity indices for 27 HIV-infected migrants, Italy, 2004–2007. Horizontal line indicates the cutoff value. ID, identification.

**Table 2:** Likely time and place of infection for 27 HIV-infected migrants, Italy, 2004–2007

Time of migration	Antibody avidity index <0.8 (infection acquired in past 6 mo), no. (%)	Antibody avidity index >0.8 (infection acquired >6 mo earlier), no. (%)
Past 6 mo	1 (3.7) (place of infection is undetermined)	4 (14.8) (likely place of infection is country of origin)
>6 mo before HIV testing	6 (22.2) (likely place of infection is Italy)	16 (59.3) (place of infection is undetermined)

## Conclusions

Our data confirm that many illegal migrants practice unsafe sex (low rate of condom use). These findings are worrisome if one considers poor knowledge of HIV transmission reported in our study population (*11*). Consequently, migrants are particularly vulnerable to STIs, as shown by the high prevalence rate (0.97%) for the adult migrant population, which is higher than the estimated 0.4% prevalence rate for the national population in Italy (*12*). The higher HIV prevalence rate for persons from an area (sub-Saharan Africa) in which HIV is highly endemic might reflect exposure in the country of origin or new infections in the host country. A total of 6 (22.2%) of the 27 HIV infections for which avidity index data were available were probably acquired in Italy by migrants from sub-Saharan Africa (n = 3), eastern Europe (n = 2), and Latin America (n = 1). Conversely, all 4 (14.8%) persons who acquired infection before migration to Italy were originally from sub-Saharan Africa.

Our study had 2 limitations. First, recruitment was not evenly balanced between centers. However, migrants are unevenly distributed in Italy, with the largest communities in northern Italy. Second, the study acceptance rate was only 73.6%. However, this acceptability rate is similar to rates in other studies in Europe (*13**,**14*). Furthermore, sociodemographic characteristics of our sample were homogeneous with those of other studies from the same centers and nationwide (*1*,*15*), and we did not anticipate any differences between participants and those who did not participate.

Our data show high HIV prevalence for an illegal migrant population in Italy. For persons originally from sub-Saharan Africa and, as for the native population in Italy, practicing commercial or unsafe sex were independently associated with HIV infection. A large proportion of persons had presumably acquired infections after migration. These data indicate the prominent role of social determinants of HIV infection, including marital status and living conditions. Health education and free access to HIV testing and care for the illegal migrant population in western countries is needed, particularly for persons from sub-Saharan Africa.
